# Sources of madness: investigating the post-colonial history of psychiatry in Niger

**DOI:** 10.1017/mdh.2025.10032

**Published:** 2026-04

**Authors:** Gina Aïtmehdi, Camille Evrard

**Affiliations:** CNRS, France

**Keywords:** Psychiatry, Post-colonial History, Niger, West Africa, Ordinary of Madness, Historiographical issues

## Abstract

This article is an attempt to reconstruct the history of the first Nigerien psychiatric service, and diverse aspects of the ordinary functioning of Pavillon E in Niamey (Niger): the organisation of daily life, the position occupied by *coopérant* doctors, the precise perimeter and development of practices taken from social and community psychiatry, and relationships with the outside world (families, police, legal system, the public health office).

This research allows us to rehistoricise and refine the details of a period from 1950 to 1980 which, up until now, was viewed as fixed and anachronistic. We draw on precious sources of empirical data – medical and administrative archives, students’ dissertations, oral sources – which invite us to reconsider both colonial/post-colonial (dis)continuities and the temporal caesuras in the literature or in reports from the time.

This landscape of mental healthcare appears to be more or less deeply affected by regional and international dynamics, such as the French *coopération* system, the networks of ethnopsychiatry and transcultural psychiatry, or the network of pharmaceutical groups and their subsidiaries.

Studying this service also raises the issues of the chronology and daily life of post-independence psychiatric care in francophone West Africa. Finally, our research interrogates the intellectual partitions between reforming disalienist movements and day-to-day psychiatry, and addresses fundamental epistemological questions on how historiography can restore the balance of knowledge between them.

## Introduction

In 1980, Patrick Osouf, a French *coopérant* ^1^ doctor in Niger, writing in the well-known francophone journal *Psychopathologie africaine*, provided an alarming overview of the country’s psychiatric service, Pavillon E.^2^ In contrast with the already declining influence of the Fann School in Dakar, the main service for treating madness in Niger was portrayed by the author as a place stuck in a ‘pre-therapeutic’ age:^3^ an institution that was organised – still according to the author – on the basis of an obsolete asylum model that had its roots in the colonial period. The service, which was set up immediately after independence, was in all likelihood very far from the model ‘of a psychiatry without psychiatrists’ advocated by Henri Collomb, who pioneered a major social psychiatry movement at Fann in Senegal from 1958 – a movement in which Osouf himself was involved.^4^ This model aimed to restore a place in the community for mentally ill patients who had been excluded from society and locked up inside closed institutions. The author described an overcrowded service in Niamey which, from 1978 to 1980, catered to around 230 hospitalised adults a month, with a capacity evaluated at only 118 places in total.^5^ He thus outlined the general conditions of mental healthcare during these post-independence decades, often overlooked in the history of psychiatry literature. The present article aims to tackle this historical and clinical black hole.

This article is motivated by an initial observation: that of the near absence – still to this day – of any knowledge about the post-colonial history of day-to-day psychiatry in Niger. From the 1950s to the 1980s, a series of initiatives were launched that brought new ways of thinking about psychiatry to the African context, spearheaded by both English- and French-speaking psychiatrists, such as Thomas Adeoye Lambo and Henri Colomb, leading figures of these reform movements. Numerous accounts, studies, works, and testimonies about these transformations appeared in specialised literature or at pan-African psychiatric conferences during this period, and served as a basis for the historiography. The dominant narrative of this reformist trend reflected the pride of place in the progressive and disalienist initiatives of the 1960s and 1970s, in particular, held by French-speaking West Africa, of the Ecole de Dakar.^6^ Its exceptional status thus elided more ordinary psychiatric services.^7^ In Niger, other than Patrick Osouf’s article, only a few publications in the fields of social science and psychiatry have documented the transformations that affected the treatment of madness at Pavillon E.^8^ Reconstructing this local history provides a new perspective on the history of psychiatry in Africa, seen from below, by complicating dominant narratives of the expansion of social therapy and the introduction of local knowledge – or the modernisation and Africanisation of psychiatry – and the chronology of its spread. Going against, but also mirroring works on the reformative movements of post-colonial ethnopsychiatry and transcultural psychiatry – embodied by the Dakar School and its related literature – we also outline new avenues for epistemological reflection.

The task of defining the milestones of post-colonial psychiatry in Niger based on its main service for treating mental illness poses a methodological challenge. Inspired by the differences in sources and methodology of our respective research work and training,^9^ the present essay is located at the juncture between medical and institutional history, political history, and oral and localised history. This research follows the development of this service and its chronology between the late 1950s and the 1980s based on a plurality of sources that each offer their unique perspective and subjectivity, and whose narratives have to be stitched together. The ‘psychiatric assistance’ archives from the National Archives of Niger (ANN) mainly speak to the late colonial period, while the psychiatric, medical, and administrative archives of the service itself, especially the nurse registers, provide information on the number of patients and its evolution. We also worked with a corpus of a dozen student dissertations from the *Ecole nationale de santé publique* (ENSP, or the National School of Public Health) relating to internships carried out in the psychiatry department between 1974 and 1992. These documents by student nurses and social workers are invaluable as they provide detailed, sometimes highly critical, information on how the department operated during a period that lacks other sources. This is also the case for the two doctoral theses in medicine from the Université Abdou Moumouni of Niamey, theses based on several years of practising in the service in the 1980s. This paper also relies on guided interviews carried out with both former and current staff members of Pavillon E, either drawn from earlier studies^10^ or undertaken for the present project. In addition to the testimonies of Yaro Gourmandeye, the ward’s first nurse, and Sadio Barry, the first African psychiatrist to arrive at Pavillon E in 1983, we also draw on the testimonies of the professionals who worked alongside Barry or after, and who inherited their narratives. This vocal polyphony establishes an oral history structured around a chronology marked by the arrival of Dr Barry, a shift reflecting the institutional move from ‘lunacy’ to ‘modernity’.

Although the Ethics Committee of the Ministry of Public Health and of the Ministry of Higher Education and Research approved this research, thus facilitating access to resources, many details remain unclear. The present article confronts the challenges connected to the production of knowledge in a context of structural disinvestment in institutions, without omitting the political,^11^ practical, or material^12^ constraints that limit access to already disparate sources. We hope to confront the difficulty of integrating the local history of the service with the national history of Niger, given how little we know about political dynamics that impacted Nigerien healthcare during the first two post-colonial regimes.^13^ As such, the present study is the result of painstaking work to reconstitute the development of the service between independence and the 1980s, and it deconstructs the dominant historiographical narratives and the ‘classic’ narrative of the ‘Barry Revolution’ propagated by subsequent generations of psychiatric personnel. Based on these sources, this article establishes an outline of the ordinary conditions of treatment in this psychiatric service during the post-independence period. We also think more widely about historiographical production on West African psychiatry by re-evaluating the study of psychiatric hospitals – whose evolution is punctuated by narratives of inertia and even neglect – perhaps more representative than the flagship hospitals of Fann in Dakar or Aro in Abeokuta.

In its first part, this article highlights a disconnect between the Niamey psychiatric service and the dynamics of regional and international psychiatry networks during decolonisation. We then weave in a detailed history of Pavillon E, which reveals the pavilion’s evolution from an asylum-like situation, often associated with the colonial period but which remains to be truly defined, towards a psychiatry of care. Finally, the third part analyses oral accounts addressing the ‘Barry Revolution’ as a turning point for the institution. We thus take seriously claims of a break from a period before the 1980s, subjectively considered as frozen and timeless.

## Niger and the networks of psychiatry from 1950 to 1980

Before we attempt to reconstitute the history of daily life in Pavillon E, we must locate it within the regional and global history of African psychiatry during decolonisation. The former French military territory and then colony of Niger was not included in the territorial federation of French West Africa (*Afrique Occidentale Française* – AOF) until 1922. Thereafter, the colony developed under the supervision of federal health and social policies until its independence. Its late inclusion in the federation and its landlocked position increased the backwardness of colonial policies that were already not very dynamic from the start.^14^ In terms of mental healthcare, the absence of any coherent federal policy continued through to 1938, when a specific, appropriate legal framework was created, setting up a psychiatric care service in AOF, which however would not be of much practical consequence.^15^

The first psychiatric service in Niger slowly took shape at the end of the 1950s. It may be that it benefited, as was the case in Senegal,^16^ from funding from the *Fonds d’investissement pour le développement économique et social* (FIDES), whose second plan (1953–1958) financed several psychiatric institutions in the region. Compared to Algeria or Madagascar,^17^ or to the psychiatric institutions of certain very close British colonies,^18^ this was very late in the day. Historiographical works on progressive movements in psychiatry and their connections on the continent^19^ at the turn of various independences have particularly highlighted the initiatives of Thomas Adeoye Lambo in Abeokuta^20^ or of Henri Collomb in Dakar.^21^ But in spite of the geographical and cultural proximity of Niger, one struggles to identify how the future Pavillon E might have fit into these dynamics. On the contrary, several indicators reveal divisions between the movements of ethnopsychiatry, present in Niger as early as 1954 because of an interest in spirit possession, and day-to-day psychiatry, which was nearly invisible. This situation was not unique for this period, where a majority of psychiatric services in the sub-region remained on the fringes of these reformist movements.^22^

While the Dakar School was at the heart of the first African networks for discussion and research in the field of psychiatry in the 1960s, in connection with their Anglophone predecessors, and, over the years, practitioners of all nationalities who had trained at Fann spread out to numerous services throughout the sub-region,^23^ Niamey seemed to remain detached from this movement during the first decades of existence of Pavillon E. The traces left by the psychiatrist Charles Pidoux^24^ allow us to analyse how local and international structures related to each other. He seems to have worked in Niger as early as the 1950s, and then to have taken on the management of Pavillon E without it being possible to say at exactly what moment,^25^ and he is very present in the archives of Pan-African psychiatric conferences between 1958 and 1968.

His publications in *Psyché*, a Christian psychoanalysis journal, in 1955,^26^ clearly reveal his interest in ethnopsychiatry applied in Niger in particular (and almost exclusively within songhaï-zarma society).^27^ Inspired by the discovery of local sociocultural institutions – or, to use his own terms, of a ‘pre-scientific’ knowledge about madness^28^ – Pidoux, through his research and clinical experiments, took part in feeding into discussions about the relationship between ‘madness’ and ‘culture’. Developing a Nigerien clinical case, he examined the resources of local therapeutic practices, and suggested that he was already known and respected by the region’s *zima* (healers). Several years later, he played an important role during the three inaugural meetings of a continental psychiatric network that aimed to build the foundations of an African psychiatry,^29^ which were organised in 1958 and 1959 in Bukavu, Brazzaville, and Antananarivo. He was involved in preparing these, then was their co-rapporteur, and gave several presentations. The way he is described confirms his academic background: we successively read that he was a ‘sociologist and psychiatrist with experience of Africa’, an ‘ethnopsychiatric director at the CNRS’, or a ministerial technical adviser. The areas he investigated were varied, ranging from the psychosociological aspects of possession rites to those of migration, via ‘traditional education’, encephalography, or religious practices.^30^

1965 saw the foundation of the *Société de psychopathologie et d’hygiène mentale de Dakar* (Dakar Psychopathology and Mental Hygiene Society), whose works and network built on those of the new journal *Psychopathologie africaine* and the beginning of post-independence Pan-African psychiatry congresses. At this moment, we meet Pidoux again, both publishing in the aforementioned journal in 1967, and taking part in the Dakar symposium in 1968 (the second after Abeokuta, Nigeria) as a representative of Niger. Here again, he gives numerous presentations.^31^ We then lose track of him during the following gatherings, in which he does not seem to have taken part, in particular the 4th Pan-African Psychiatry Conference that was held in Abidjan in July 1975, or the 7th International Seminar of the African Association of Psychiatry in Yaoundé in October 1977.^32^

Thus, while Niger was represented, first conceptually and then officially by Pidoux during these years that saw the structuring of the field of post-colonial African psychiatric research, the only hospital service in the country that took in patients suffering from mental illnesses does not appear to have benefited from these changes. In the parts of his writings^33^ that we are still able to read today, nothing specifically refers to psychiatric care in Niger, or even to Niamey Hospital,^34^ as if the ethnopsychiatric approach had no place within this institution. However, discussions of these international meetings are very often connected with the fundamental issues that also affected Pavillon E in its early years: how to train African psychiatrists, the difficulties encountered by the discipline in ‘developing countries’, sociotherapy, therapeutic villages, etc.

This disconnection seems to become clearer when we examine the concrete situation in the service in the middle of the 1970s, when Pidoux was at its head. The dissertations written by students of the *Ecole nationale de santé publique* who took part in internships under the supervision of the head doctor, express – with a freedom probably connected to their position within the institution – the fact that the only psychiatrist in this service was often absent, being notoriously busy:

The doctor does not always see patients when they are admitted. Nevertheless, he sometimes carries out visits, prescribes treatments and does receive some patients in his consulting room. One cannot precisely determine when the doctor will carry out a visit. Most of the time the head nurse has to very much insist and take the doctor to see all the patients in their beds. This is mainly due to the fact that the doctor, aside from being responsible for the psychiatry service, is also in charge of providing expert assessments of criminals as requested by the judicial authorities. He is often invited to conferences, which does not leave him with enough time to take care of psychiatric patients.^35^

In the second half of the 1970s, transnational psychiatric networks, encouraged by the WHO, underwent a new development: from regimes of experts mainly focused on research and training, these movements tended to shift towards a mode of political advocacy which, in particular, encouraged the definition of national programmes in favour of ‘mental health’.^36^ However, it remains difficult to define the contours of this development in Niger, even if it seems that the psychiatric service did start to undergo such a process under the military regime of Seyni Kountché.

The latter seized power in Niamey in April 1974 following a putsch which no one seemed to have anticipated:^37^ the Supreme Military Council carried out a great populist clean-up of what they denounced as corruption in the Hamani Diori regime, which had been in power since independence and was on its last legs. S. Kountché’s long stint in power, through to his death in 1987, in its turn developed into an authoritarian regime with successive waves of repression, even if it is commonly viewed by the population as a period of revitalisation or even of modernisation, which supposedly also included mental health.^38^ Several accounts insist on the desire for change that is connected with the Kountché period, one of the tangible elements of which was the project to create an independent psychiatric hospital on the outskirts of the town: developed around 1975, this project was included in the five-year plan but was not realised for financial reasons.^39^ The way in which Osouf describes the project and its definition illustrates the conflict between the two views of psychiatry, which largely co-existed in the sub-region at this point:

The projects in question (Project 1977) only leave little room for spaces for institutional or group psychotherapeutic care, for spaces of creativity, for an openness to the invention of harmonious and specifically Nigerien formulas. Its originators rely on a classical tradition, which itself has long been called into question in the West, with no appreciation for the country’s cultural and human realities; their projects inevitably end in the retrograde, asylum-based institutionalisation of madness.

Furthermore, Dr Sadio Barry explains that: ‘Kountché demanded modernisation. He asked the WHO how to proceed, and an expert (…) came to Niger. A Mauritanian, Dia. He wrote a document in which he said that what we were doing in Niger was anything but psychiatry and that the service was a menagerie: “a pigsty”. This very much upset the president’.^40^ We see here the tensions at work between the desire for modernisation and the dissonances between political government and government by ‘experts’. The fact remains that the assignment of a taskforce, sent over in the WHO’s name, and directed by the Mauritanian psychiatrist Al Houssein Dia to advise the government of Niger regarding the development of a national mental health service, took place in this context.

The ‘Dia Report’ is the missing link that might have allowed us to better relocate the history of the service within the context of regional interactions and to understand the efforts by the AFRO Group within the WHO to encourage co-operation^41^ in the field of mental health. It could also have helped to analyse relations to the Dakar School, since Dia too had been trained at Fann a few years earlier.^42^ Although this report has disappeared – even from its author’s own archive – we know for certain that the assignment did in fact take place in 1983.^43^ He recalls the discussions that were held with the Nigerien committee responsible for developing new solutions by insisting on his radical opposition to the project of building a new hospital:

The Nigeriens wanted to build a new hospital outside the city; I said don’t make a prison over there, there’s no point! Try to humanise this service, to decentralise it, don’t put all the patients in Niamey, that’s not what they need. When they come for a consultation, all you need is a week of treatment, then you send them back to their families with some medicine and they’re fine.^44^

He reports having been extremely shocked by the state of Pavillon E, in particular by how overcrowded it was and by the staggering number of chronically ill patients, but also by the living conditions of patients locked up in the ‘*cabanon*’ or ‘hut’: naked, on the bare ground, having sometimes been kept there for decades, and facing problems with the supply of basic medicines. He also mentions that a French psychiatrist was also present, but that he seemed to him to be ‘very distant’, as if uninterested in these issues.^45^ It is unlikely that this was Osouf, who like him had trained in Dakar^46^ and with whom he would have had to communicate, but we do not know at what date Osouf left Niamey.^47^

These lines have inspired the hypothesis that Niger – or at least the psychiatric service of its National Hospital – was considerably out of step with prevailing networks of thought on an emancipated and emancipating African psychiatry, giving the impression of a graft that did not take. The criticisms formulated in the early 1980s reveal something resembling a yawning gap between the situation in Pavillon E (or in the Kountché government’s projects for mental health services) and the progressive movements arising out of the Dakar School or Pan-African conferences. They also give the impression that time stopped in the service, blocked in a kind of ‘asylum-based’ psychiatry that is often somewhat quickly associated with the colonial moment and which nevertheless remains to be defined. Going back into the normal operation of the service, its development and transformations, its daily life, in spite of the grey areas due to rare and fragmentary sources, allows us to resituate Pavillon E within a more finely grained temporality, and to reinsert it into the complexity of practices that were applied at the service during the 1960s and 1970s.

## Daily life at the service: an exploration (1952–1983)

Towards 1953, the idea of psychiatry made itself felt in Niamey, and mentally ill patients were treated in asylums where they were handcuffed because they were viewed as dangerous animals. At this time, there were cells in which women would be locked up in the central dispensary. The men’s asylum occupied the space which is now the kitchen in Niamey Hospital. We should also note that there was no psychiatrist, which suggests that these patients were not treated. They would just be calmed down with Phenergan if they got excited, since psychiatric care was not provided in Niger until 1953. Three years later, Doctor Pidoux brought along an electroshock machine (1956). The new service was not launched until 1960, under the directorship of Doctor Tchelle, who was a general practitioner with psychiatric training.^48^

Challenged by Osouf’s article, we here intend to undertake the work of reconstituting the infrastructures, functioning, and staff of the service, from the 1950s through to the arrival of Sadio Barry in September 1983. Elements gleaned from ENSP dissertations (mainly internship reports written by nursing or social work students), the hospital’s admission registers, as well as reports provided by professionals, allow us to trace the contours of the service. In so doing, we reveal a more nuanced picture, with the opening of a dedicated service at the turn of independence, which in spite of all its limits provided the conditions for a fundamental break with the previous situation. Yaro Goumandeye, the first psychiatric nurse in the country whose career among the ‘madmen’ extended from around 1953 to 1982, was a pivotal figure of mental assistance in Niger throughout this period. The pioneering work of F. Saint-Girons, founded in particular on extensive interviews with this now deceased witness, allows us to reinterrogate the chronology of this care and its nature: the service was created at the turn of independence, but did not break with the strategy of locking up mentally ill patients.^49^ Nevertheless, Y. Goumandeye reports a variety of practices based on a pragmatic vision for the reinsertion of patients into a social web, which can *in fine* be viewed as therapeutic – despite the limitations in the care provided that can otherwise be observed. Furthermore, the service had routines, activities which gradually became established, and its functioning was integrated into the wider logic of the National Hospital – with all of these being elements which also ‘held’ and ‘contained’ the patients. Reconstituting this history of the daily life^50^ of Pavillon E, through its first years of existence, allows us to start asking the question of what constitutes a transformation.

At the start of the 1950s, Niamey had, as well as the hospital created in 1922, a ‘central dispensary’ connected to several healthcare infrastructures on the periphery of Niamey and working under the supervision of the French army physician directing Niamey Hospital. The most ‘dangerous’ or undesirable mentally ill patients were then treated in a few rare closed spaces connected to the dispensaries, whose aim was to keep them locked up.^51^ The control of ‘madmen’ in urban spaces was structured, as it was elsewhere,^52^ around police actions targeting populations that were viewed as undesirable, which were regularly ‘rounded up’ – but there was a definite lack of dedicated spaces in Niamey: in 1952, we can find only two spaces that were able to receive ‘lunatics’, a ‘hut’ at the dispensary and an ‘isolation room’ at the hospital.^53^ Yaro Goumandeye started to shuttle back and forth between these two places of relegation for the treatment of ‘madmen’ from 1953.^54^

In terms of infrastructure, apart from the two places mentioned in 1952, one of which was reserved for women and the other for men, the system’s capacity changed around 1955: during an extension of the hospital, a ward was built on the first floor of the building closest to the main entrance^55^ in order to cater to mentally ill patients; and in parallel, Pavillon E was built, a general medicine service for various categories of civil servants. This then became a psychiatry pavilion between 1960 and 1962, with the space being partitioned: the ground floor was reserved for mentally ill patients and the hut, and above it was the general practice for civil servants.^56^ During the second half of the 1960s, Pavillon E was extended and divided into two parts: one for men and one for women, who were then transferred to the ground floor: it was necessary to prevent sexual abuse and, more generally, patient sexuality.^57^ The space reserved for the general hospitalisation of civil servants now catered to a new type of patient:^58^ common law or political prisoners, who were brought here from prison when they required medical care (usually of a non-psychiatric nature).

The presence of undesirables who had been ‘rounded up’ on the street, or of political prisoners, near the psychiatric service, reveals,^59^ subsequent to colonial policies, the governmentality of madness and the post-colonial decisions made in terms of political and social rights. Niger became independent in 1960, following a process close to that undergone by other colonies in the group formed by former French West Africa, and which was in particular rife with political manoeuvres^60^ through which France did everything to enable the access to power of the elites with whom it had good relations.^61^ This was the case for the first Nigerien president, Hamani Diori, whose authoritarianism was reinforced by the consolidation of the (PPN-RDA) party-state and the ferocious repression of the *Sawaba* opposition movement.^62^ The regime worked in harmony with Paris, which for many years provided it with numerous experts in all the fields of administration, healthcare, defence, and security. This so-called moment of national construction is the one for which we most significantly lack documentation about the psychiatry service and, more broadly, about the history of healthcare in Niger.^63^ Nevertheless, we can identify a form of continuity of practices with the colonial period, or even of reinforcement of these practices, with the creation of a dedicated psychiatric service: the ‘undesirables’ who were regularly removed from the public space for reasons of public order, and the prisoners who had to be kept locked up and monitored while they received medical care, were associated with hospital-based psychiatric practice and stigmatised this practice. Osouf again writes about the presence of prisoners in the service in 1980, which ‘consecrates the very detrimental assimilation of hospitalisation for mental illness with penal incarceration, both in the eyes of the public authorities and general public opinion’.^64^

Another central continuity after independence was Goumandeye’s involvement as a native nurse. Given the inadequacy of the healthcare system and the lack of local staff, he already enjoyed a relatively considerable autonomy in relation to French military doctors in the colonial period,^65^ and he continued to be the main daily figure for patients and staff when he was supporting psychiatrists who now tended to be *coopérants.* Trained as early as the 1940s in the first institutions aimed at native healthcare staff,^66^ the career of Goumandeye is striking. He became the first psychiatric nurse after specialising at Fann around 1960, as well as having a family heritage which, according to him, allowed him to ‘treat diseases of the spirit’.^67^ The length of his presence at Pavillon E makes him a central figure of post-colonial psychiatry, since he retired in 1982 after having been a major figure for many years – a genuine kingpin of the service, the doctor’s right-hand man, head of staff, administrator of reserves. Following the official creation of the service under the guidance of Dr Tchelle,^68^ the system structured around the duo of a Nigerien nurse and a French doctor endured. Upon returning from Dakar, Goumandeye started to work with the first in a long series of ‘*coopérant*’ psychiatrists, who he called Dr S. Up until the arrival of Patrick Osouf at the end of 1978, it is difficult to establish a precise chronology of the presence of these doctors and their identity: S., Charles Pidoux, Alain Bertrand, and other anonymous figures passed through the head office of the service during the 1960s and 1970s. For a long time, this office remained by the entrance to the hospital, which reinforces the impression of there being a disconnect with the daily life of a service that quickly became overcrowded and which was becoming increasingly stigmatised.

The organisation and daily life of the service largely appear in the dissertations of student nurses from the second half of the 1970s: they are particularly critical, which suggests that there was probably a consensus among the staff regarding certain practices that were viewed as inhumane and associated with the 1950s, in particular for ‘agitated’ patients who were locked up in the ‘hut’. However, no cause or responsibility is pointed out – we just observe a lack of resources, expressed through the use of the still powerful rhetoric of underdevelopment. The main criticisms are targeted at the lack of qualified staff: only one doctor, a head nurse who was the only specialised nurse, and a number of nurses that increased from two to five between 1974 and 1979, as well as around ten subordinate staff ranked as ward housekeepers, unskilled workers, caretakers, and ‘boy cooks’. They also point out the dilapidation of the premises, the dirtiness, the dangerous electrical system, the defective toilets, but above all, the poor quality of the food given to the patients in largely insufficient quantities. Mealtimes are particularly observed and criticised: there are not enough vegetables, fruit, or meat, and the rations are too small. Foreshadowing the observations of the Dia Report, a student writes in 1976:

The distribution is carried out in arduous conditions. (…) The boy is not often able to identify all the patients who have been served. General hygiene measures are not complied with. The cups have become black and are full of holes. They leak a large quantity of foodstuffs. Most patients, in particular those of the men’s hut, do not always receive their ration. Some of them lick the ground if sauce is poured onto it. (…) The patients from the hut have neither bed, nor mattress, nor mat, nor covers, nor showers or toilets, and they are partly deprived of food and distractions.^69^

In a context of constant overcrowding, the impossibility of providing care was then passed down in a domino effect: the psychiatrist head doctor had to treat the patients as well as the prisoners, but he only rarely saw them, since there were too many of them; the head nurse was responsible for supervision and administration as well as for providing care in the ‘hut’, and also had to monitor treatments, make observations, and signal issues to the doctor if need be; nurses worked in three 8-hour shifts and the head nurse during the day only. Nurses were sometimes left alone during the night with almost 200 patients; they therefore had to be able to rely on the ward housekeepers. The daily life of patients in Pavillon E, most of whom, it is specified, were brought in by the police during this period, and more rarely by their families,^70^ was even more difficult for the category of so-called ‘indigent’ patients, who had no known parents, or whose families were not able to provide any additional support.^71^ A particularly vehement dissertation from 1979 even mentions that, unlike those who received their care in bed, these patients were treated in a common room, with the same syringe and often the same needle for injections – and by a ward housekeeper.^72^ This author concludes:

There is a genuine sloppiness, a lack of professional conscience in this pavilion. The patient is viewed as an inanimate object, and does not enjoy any rights concerning the respect of personal dignity, for it is indisputable (*sic – the author most probably means “unacceptable”*) for people without any training provide care to patients. Of course this contributes more to destroying than healing the patient.^73^

The admission registers for Pavillon E that have been partially retrieved for the periods 1966–69 and 1975–79 allow us to define some general trends in hospitalisations during these first two decades of the service’s existence. The number of people admitted to psychiatric hospital per year increased considerably and regularly, going from an average of around 400 in 1967 and 1968, to 500 in 1975, and reaching 650 in 1978.^74^ But this does not reveal how many patients were present at the same time in a service that only included around a hundred beds. Student dissertations confirm the overcrowding which Osouf condemned: at the beginning of their internships, they mention 202 patients in 1976, over 230 in 1979, 194 in 1980, and 220 in 1981.^75^

While Osouf wrote the first critical scientific article on psychiatric care in Niger, students on internships in the 1970s had uncompromisingly documented the living conditions of patients and staff. When Osouf arrived, the service was more overcrowded than ever and staff numbers were extremely low: a total of 8 people for 250 patients, he writes, which amounted to – due to the timings of shifts and breaks – an average of one nurse for 50 people.

The medical situation in the psychiatry pavilion in Niamey appears critical: it is essentially connected to the overcrowding of a service that no longer has any therapeutic function. This overcrowding has led to a progressive paralysis of the medical team’s efforts. It has become impossible to monitor patients, which is harmful to the efficiency of care, to therapeutic action and to the safety of individuals. Medical staff, who already lack motivation to work in this difficult specialty, no longer control their actions and are both physically and psychologically overwhelmed.^76^

The doctor’s opinion on the quality of the work carried out and the possibility of providing therapeutic care is final. However, the account of a psychologist doing an internship in the service at the same time, Patricia Brossat,^77^ as well as that provided by head nurse Goumandeye^78^ throughout his long career, allows us to glimpse some instances of genuine care work being carried out. We see here the possibility of bonds being created, in particular with chronically ill patients, kindnesses that go beyond the walls of the hospital, and an ability to allow ‘traditional psychiatry’ practices^79^ to exist alongside sometimes punctual admissions to the service. Collective activities, already referred to as ergotherapy,^80^ and which allow certain patients to make artisanal objects that are then sold at the National Museum, thus generating a little revenue for them;^81^ organised days of collective cooking in the women’s space, thus recreating the more familial atmosphere of a large traditional household;^82^ and above all, the largely informal and constant nature of the relatively free communication between all players in the service, from doctors to patients, through the head nurse, interpreters, or ward housekeepers and caretakers.

Patricia Brossat, while she does not shy away from describing difficult scenes that also reflect some terrible conditions, in particular those of being locked up in the ‘hut’, ultimately almost paradoxically reveals the humanity and care that are also part of daily life in the service: people communicate and sometimes even heal.^83^ Although they had already been implemented for years under Goumandeye, practices resembling sociotherapy seemed to consolidate during Osouf’s time at the service – albeit carried out with whatever means were available and despite the overcrowding of the service. Dissertations by ENSP students largely bear witness to this from 1981, which was also the year that saw the arrival at the service of its first social worker, Georgette Guillemin.^84^ All of this thus outlines the subtlety and diffuse aspects of the transformations underway during this second decade of existence of the Niamey psychiatric service.

Psychiatric logics that might seem contradictory co-exist side by side, reflecting a daily life in evolution. Logics of solidarity and care can thus emerge in a generally rudimentary context driven by asylum logics. However, as we will see, the history of the service, conveyed locally by different generations of caregivers, is less nuanced. It highlights a radical transformation of the service under Dr Barry, clearly marked as a ‘before and after’ between the asylum model and the advent of modernity.

## The ‘Barry Revolution’: oral history and historicity of Pavillon E

Pavillon E’s complex history is also the object of oral history deployed in the informal transmission of knowledge between service personnel. This history is marked in particular by a ‘revolution’, a crossing point towards a new era of psychiatry in Niger. (Re)constructing a historiography of the service implies indeed examining the local discourses produced and transmitted by medical and hospital staff which underwent a break with the arrival of Dr Sadio Barry. While, in a relatively uniform manner, the population viewed Pavillon E as *the* symbolic location of ‘madness’ in Niger,^85^ the generations of healthcare workers who worked here one after the other, for their part, became acquainted with more complex and detailed chronological narratives. Reports from staff members from this period constitute an essential resource to be collected, reproduced, and interrogated as a source. It is also a constructed knowledge conveyed by Dr Barry and his nurses, Awa Sylla and Idi Kona, that needs to be situated. The chronology in these oral histories seems to be relatively stable and constructs the ‘revolution of Pavillon E’ led by Barry and his team as a fundamental break in the history of the psychiatric service. This turn, viewed as ‘modernist’ by these staff members, radically defined a before and an after ‘Barry’, a transition from the age of barbarity instead of care and a profound recasting of the relationship of local psychiatry to mental illness under the belated influence of Fann, as embodied by Barry.

This psychiatrist, recognised as *the* figure who reformed psychiatry^86^ in Niger both on a local scale and within West African mental health networks, more assertively exported certain principles of the Dakar School, which up until that point, Pavillon E had probably only slowly started to benefit from. Trained at Fann in the 1970s in medicine and then in psychiatry, and originally from Guinea, Dr Barry emigrated to Niger for personal reasons, and definitively settled there in 1983. He became the first African psychiatrist in the country, and adopted the position of spearhead of the sector in a context where, like a mirror of Dakar, here everything still needed to be done and (re)thought. Accounts of this pivotal moment, provided by Barry himself and by the staff working between the end of the 1970s and the decade of the 1990s (head nurses of the service, nurses, caretakers), and then taken up again by those who came after them, present this turning point as a movement of massive transformations. They oppose this to the ‘dumping ground’ that pre-existed up until this point,^87^ where ‘madness’ was synonymous with bestiality, with being locked up in the ‘hut’, where the ‘madman’ was subjected to retrograde mental health treatments. This earlier period is viewed as anachronistic and is conveyed as such through the crudity of the treatments inflicted on mentally ill patients, which are supported by the traces of an architecture inspired by the carceral aspect of (post-)colonial psychiatry:

It was our hut. It was hermetically sealed. And there it was cells. Like prison cells. There, and this is where the food went through and that is a waste pipe for faeces, for excrements, for food. It was completely closed.^88^

The hut and its vestiges – a huge metal fence enclosing the space – bear witness to the imprint of the poor treatment inflicted on patients who were described as ‘madmen’, ‘animals’, and ‘beasts’ to underline the whole dehumanising character of this logic of the asylum.

The reformers’ accounts, through their rhetoric and the vocabulary they employ to refer to the so-called ‘asylum’ period, re-emphasise this caesura. Indeed, they always frame as a chronological counterpoint their descriptions and comments on a series of reforms aiming to liberate the ‘madmen’, at relieving the service from an overpopulation of indigents, at favouring, instead of surveillance, medicalised care similar to that provided in the hospital’s other services. The before and after are described in all aspects – architectural, medical, and organisational – highlighting how and by what means this anachronistic service was transformed into a psychiatric pavilion that was worthy of the name.

Through the narratives provided by staff members, we see that this ‘revolution’ is based above all on a principle of humanisation of psychiatric care and, as such, of the patients themselves. The ultimate act was the liberation of the ‘madmen’ from the hut, which gave back a common humanity to both caregivers and patients. The heart of this reformative movement unfolded around categorically challenging the idea that mentally ill patients were naturally aggressive. Although a cross-reading of oral and written sources rather forces us to temper the radicalness of this turn by taking into account the logics that were already at work before Barry’s arrival, the fact that these discourses represent institutional violence as the tipping point for this new local psychiatric policy shows the importance of this ideological change, in which treating bodies through confinement became obsolete:

That was a change. They were locked up, all those who were aggressive. And there were family members who requested it even. They didn’t want to see them outside because they were aggressive. They would kill each other too. How many times did we open the cell in the morning and take out corpses. It was nasty between them. It was in what are now the offices. […] There were some who were less aggressive and who were in the patio. […] It was the hut and people thought that by opening all the doors it wasn’t serious and we tried and it worked. And it continues to work. We decided progressively, little by little.^89^

These changes were pushed forward by certain principles from reformative movements in post-war psychiatry and in post-independence social therapy, and were situated at a crossroads with the increasing influence of global mental health.^90^ They were faced with some resistance, but also aimed to reintegrate mental health patients within the (human) community:

I wrote a letter to the Minister. I said that I want to humanise the psychiatric service but it will be an open service, not an armoured service like you see in France with lots of doors. I said no! That’s what I learnt in Dakar.^91^

This process of opening up closed spaces – dating back to 1985 for the hut, and unfolding more progressively through to the end of the 1990s for cells reserved for common law prisoners – was in fact accompanied by a reinforcement and transformation of the medicalisation of medical practices. While the practice of ‘biological’ psychiatry was already germinating in the service, Barry and his medical team accompanied and supported the liberation of the patients in the hut through an institutional openness to neuroleptic molecules offered and supplied by French laboratories. Like with other post-colonial contexts, the logics of sociotherapy were in fact supported by a therapeutic reform^92^ which locally enabled a control over patients that was necessary for the wider process of relieving the service:

For the neuroleptics it was Largactil, Chlorpromazine, Nozinan, Halopéridol… as a corrector we had Artane or Parkinane. And then there were injectable, depot neuroleptics. Yes because we didn’t want to get into pills […] because the patients did not accept drugs easily, so we started with a depot neuroleptic and then arrived at Modecate. This was before the 1990s, with the Sanofi laboratory. They had medical delegates who were travelling everywhere. […] The one we didn’t use much was Haldol because it’s expensive. So we had the neuroleptics. We had the anti-depressants, mainly Anafranil, Paroxetine. […] We had a whole range of products so that the poor patients, when they went home, we gave them a provision of them. We gave patients up to three months’ worth of a product.^93^

The introduction of new pharmacological treatments, in particular extended-release neuroleptics known as ‘depot neuroleptics’, also supported a process of reorganising care. A recasting of the mandate of psychiatry was at work in this movement. The apparently more assertive resistance against forces responsible for social control and the monitoring of undesirables (prisoners, dangerous madmen, indigents, vagabonds) appears in these accounts as one of the main motivations for a pitched battle against the official authorities, at the level of the hospital, of police services, or of the government of Seyni Kountché up until 1987:

The police no longer picked up people who didn’t have any mental health issues. They used to pick up tramps. That stopped and it was discussed with the chief of police and the hospital authorities at the request of the head of the psychiatric service. It didn’t come from the hospital administration. It came from us, who were managing the service on a daily basis. It was Dr Barry who reported this complaint to hospital management and to the authorities. And really it stopped. Everyone knew about it: the caretakers, the healthcare staff. When the police brought someone in, really, you didn’t take them! And if there’s a problem you call your manager. We were given instructions. When they came, we told them to leave again with their tramp.^94^

The positioning of staff members during this period in favour of the medicalisation of psychiatric care led to a transformation of connections to external services – in the hospital, police, and penitentiary systems – as part of a process that is in fact still at work in the contemporary period.^95^ Nevertheless, the decades of the 1980s and 1990s seemed to considerably contribute to a transformation of the views of psychiatry at the hospital level via the institutionalisation of day-to-day practices which up until that point tended to be the result of isolated initiatives.

As such, the family occupies a central place in this reform. Once more influenced by clinical standards in Fann, where the role of a patient’s relatives in hospital had become central to their care,^96^ this new generation of staff established the rule that a family member – an ‘accompanier’ – must be present during the patient’s stay in hospital. Beyond clinical dynamics, this prescription avoided the patient being left entirely under the responsibility of the service, in order to support relieving the service for chronically ill patients, while constituting an additional resource against the service’s shortcomings:^97^

It was very difficult. […] We said to them […] the habits you have are no longer relevant. There are new rules. You come with your relative and you stay with them. You want to leave, you leave with them. […] We are disengaging, we’re not responsible if something happens to them. Because we don’t have enough human resources to care for them. It has happened that families leave. It still happens, even now. There are some who say they’re out of provisions and ask for permission to go and fetch some money to pay and […] we don’t see them anymore! It’s over! There are some who leave, but there are some who stay. And there are some who come but who don’t want to stay. So they come at night, they see the patient and they leave again. There are even some who come, see the guard and give him little gifts.^98^

As well as using these new rules to regulate the presence of clients in the psychiatry service,^99^ the team’s activities also aimed to make the service more hygienic. On this subject, eyewitness reports describe a process of cleaning up premises and bodies and, it also seems, symbolically cleaning up the dirt of the previous decades and the forms of ignominy still associated with them to this day:

These are mentally ill patients. I know the service didn’t have a bad reputation, no! But the only image there was this dirtiness. It was dirty when we got here. It was very dirty! We were different people really, completely different. We are! [*she laughs*]. […] People thought we were almost superhuman even though we were never attacked. I was never attacked. Never! However nervous the patient was. So that it surprised people that we were able to put up with this dirt, that we were able to put up with these smells. There were smells. How did we live? How could we eat over there? How could we live? Nothing could happen to us, we were superhuman.^100^

This process of creating a distance from madness in the asylum was actualised through ‘crackdowns’: the cleaning of spaces with soap, as well as of the patients staying in them, was directly correlated to the ‘(re)birth’ of Pavillon E:

I looked for a keg, a barrel […] which I put on the fire. I washed it and we cut up 4 barrels like that. We put them on the fire. And I completely undressed the patients to boil their clothes. And there was dirt. When I left for home my wife would say to me: ‘Look, you’ve got lice.’ And I asked for Benzo Chloride or something like that for bodies and we would completely shave them [*the patients*]. So the lice disappeared! And there were rich people, wealthy shopkeepers who would give me bundles of cloth which I would send to the laundry room to have made into clothing. The Red Cross also gave me old clothes so I could change the patients’ clothes. And so we were so completely and utterly successful that the media seized on this to sing the praises of Pavillon E. They talked about a revolution in Pavillon E.^101^

Staff members’ reports agree on defining the Barry period as the starting point of a major change in the way mentally ill patients were cared for in Niger, within a landscape that was seen as lacking in solidarity and which up until that point had tended to go against the principles defended by the avant-garde. Through this process, Niger symbolically became part of this movement of social and community psychiatry. However, Dr Barry and his team also took this approach because they were disillusioned with realities that could not be denied: the aim was above all to turn Pavillon E into a medical service like any other. Reports from the time are important to follow the content of ideological and clinical changes that left a more visible mark on the landscape of local psychiatry at the time – even if it already provided fertile ground for these institutionalised transformations. But this period, which was then collectivised and transmitted to later generations, also acquired an obvious symbolic as well as performative force: that of bringing Pavillon E into ‘modernity’ through these narratives.

## Conclusion

As we come to the end of this attempt to reconstruct the history of the first Nigerien psychiatric service, much information is still needed about many aspects of the ordinary functioning of Pavillon E: the organisation of daily life, the position occupied by *coopérant* doctors, the precise perimeter and development of practices taken from social and community psychiatry, relationships with the outside world (families, police, legal system, the public health office). The history of this psychiatric ward, which is probably more representative within francophone West Africa than more commonly studied services such as Fann, is still a work in progress. The present outline is intended on the one hand to provide an invitation to continue the work that has been started here,^102^ and to extend it to other psychiatric structures, to other ‘huts’ or services providing care to mentally ill patients which still today occupy a very marginal position in the literature, be it in the clinical field or in the social sciences.

As this article draws to a close, many nuances have been revealed, both at the level of the fine chronology of the service and at the wider scale of its place within continental movements in the history of psychiatry at the turn of decolonisation. This research allows us to rehistoricise and refine the detail of the period from 1950 to 1980, which up until now was viewed as fixed and anachronistic, by drawing on precious sources of empirical data. It also invites us to reconsider both colonial/post-colonial (dis)continuities and the temporal caesuras that go without saying in the literature or in reports from the time. The lack of accessibility of certain sources prevents us for the moment from more clearly defining certain structures at the level of the hospital, of the city, or of the government. But the documentation we have used allows for a finer chronologisation within which the logics of asylum madness are reinforced during this period, before they progressively merge with other logics of care during the era of global health. This mental healthcare landscape appears to be more or less deeply affected by regional and international dynamics, such as the French *coopération* system, the networks of ethnopsychiatry and transcultural psychiatry, and the network of pharmaceutical groups and their subsidiaries. These dynamics were implemented in contrasting ways, depending on the clinical spaces.

Studying this service also raises, with particular acuity, the issue of the chronologisation and daily life of post-independence psychiatric care in the countries of francophone West Africa. The literature on this period largely examines the development towards an integrated and community-based psychiatry through a multitude of clinical and research initiatives, at the level of services and carried out by various figures, be they French *coopérants* or African doctors. But there are not many works that examine daily life in the psychiatry departments or the ordinary course of how care was provided – and departments that first appear to be on the margins of these dynamic spheres of mental health have been particularly neglected. This study of Pavillon E hopes to shake up this overly hasty judgement, this historiographical imbalance, in order to rather direct our gaze towards those places that precisely appear as the most common and familiar to populations. We must deal here with a psychiatry that has at least two tiers during the post-independence landscape, in which carceral approaches to madness endure or are even reinforced, concealed by the spread of clinical initiatives. We must then interrogate the profound intellectual partitions between reforming disalienist movements and day-to-day psychiatry, while reevaluating the place and role of individual tutelary figures, or of moments of ‘revolution’.

The case study presented here is probably more representative of post-colonial African contexts than the literature allows us to see. Even if it is difficult to study the history of the Pavillon E in comparative perspective with fragmentary sources, our effort also suffers from the way in which experiences of modernity and ‘progress’ undergird dominant ideological frameworks. Indeed, the intellectual aspirations of historiography have taken it away from the study of other institutional and clinical experiences and their everyday life. Hypothetically, this lack of interest in the history of an ordinary psychiatric unit is part of a general discrediting of the enduring frameworks and practices of colonial and post-colonial psychiatry. The fact that Pavillon E was never a space for disasylary experiments continues to stigmatise the unit. The unit still needs to distance itself from a past that is still a part of the present. This article attempts to respond to some of these still unanswered epistemological questions.

## Footnotes

1 The *coopération* system that was set up after a wave of African countries gained independence not only allowed numerous former colonial officials to be recycled as technical assistants, but also enabled young French experts to work ‘overseas’, sometimes as a substitute for carrying out their military service, compulsory at the time.

2

Patrick Osouf , ‘Regard sur l’assistance psychiatrique au Niger’, Psychopathologie africaine, XVI, 3 (1980), 249–279 .

3
*Ibid.*, 275.

4

Henri Collomb , ‘Psychiatrie sans psychiatres’, Revue des Études Médicales, 4 (1972), 295–311 . René Collignon , ‘Les conditions de développement d’une psychiatrie sociale au Sénégal’, Présence Africaine, 169, 1 (1984), 3–19 . René Collignon , ‘Vingt ans de travaux à la clinique psychiatrique de Fann-Dakar’, Psychopathologie africaine, XIV, 2-3 (1978), 133–324 .

5 Osouf, ‘Regard sur l’assistance psychiatrique au Niger’, note 3, 259.

6

René Collignon , ‘La psychiatrie colonial française en Algérie et au Sénégal : esquisse d’une historisisation comparatative’, Tiers-Monde, 3, 187 (2006), 527–545 . Emmanuel Akyeampong , ‘A Historical overview of psychiatry in Africa’, *in* E.K. Akyeampong et al. (eds), The Culture of Mental Illness and Psychiatric Practice in Africa (Indiana University Press, 2015).

7

Gina Aïtmehdi , Romain Tiquet , ‘Introduction au thème. Penser la folie au quotidien’, Politique africaine, 157, 1 (2020).

8 Fabien Saint-Girons, *Le développement de l’assistance psychiatrique et le traitement de la question de la folie au Niger* (unpublished MA thesis: Paris EHESS, 2008); Aïtmehdi Gina, *Une anthropologie de l’ordinaire de la folie. Expériences chroniques des troubles mentaux et trajectoires de soins à Niamey (Niger)* (unpublished PhD thesis: Université Libre de Bruxelles, 2021). In the field of psychiatry, let us also mention the academic work of Brigitte Leccia: *Abord de la maladie mentale au Niger : un an d’expérience dans le service de psychiatrie de l’Hôpital de Niamey* (unpublished PhD thesis: Université Paris VII, 1989).

9 C. Evrard is a historian and analyses the sociopolitical transformations of decolonisation and of the post-independence period in the Sahel through her work on soldiers in colonial and post-colonial armies (including Niger), as well as on war traumatisms and psychiatry. G. Aïtmehdi is an anthropologist and has written on the daily life of chronically mentally ill patients in Niger, in particular by examining contemporary practices in psychiatry, thus encouraging a critical study of the medical and institutional dynamics at Pavillon E.

10 In particular, from the two academic works already cited, Fabien Saint-Girons’ master thesis and Gina Aïtmehdi’s PhD thesis.

11 Starting in July 2023, the break in relations between France and Niger following General Tiani’s coup interrupted our research in Niger. We had access to the psychiatric ward’s archives before that, but the dispersal of the papers made their exploitation difficult. This interruption has meant that we were only able to write a history from the point of view of the nursing staff, and not of the patients.

12 For example, limited access to the warehouses of the National Archives of Niger for many months between 2022 and 2023, due primarily to renovations which caused electrical problems and plunged the buildings into darkness, prevented us from carrying out research into the post-colonial collections. Some inventories were subsequently lost.

13 Unable to access any political files on public health in Niger for the years 1960–1970, in spite of our research at the archives of the Nigerien office of the WHO in Niamey, in the WHO files at the archives of the French Embassy in Niger, and our communications with WHO archivists in Geneva, it is difficult for us to reconstitute and date the decisive motivations that influenced these developments.

14

Ayouba Arziza , ‘La santé au Niger’, in C. Coquery-Vidrovitch , O. Goerg , and H. Tenoux (ed.), Des historiens en Afrique (Paris: L’Harmattan, 1998), 115–126 .

15 Up until this point, AOF had still been subject to the French Law of 30 June 1838. René Collignon , ‘La psychiatrie coloniale française en Algérie et au Sénégal : esquisse d’une historisisation comparatative’, Tiers-Monde, 3, 187 (2006), 527–545 .

16 The Fann neuro-psychiatric service opened in 1956; for more details about the early days of the service, see René Collignon , ‘Les conditions de développement d’une psychiatrie sociale au Sénégal’, Présence africaine, 129, 1 (1984), 3–19 . René Collignon , ‘Folie et ordre colonial. Les difficultés de mise en place d’une assistance psychiatrique au Sénégal et en Afrique occidentale’, in Charles Bcker , Saliou Mbaye , and Ibrahima Thioub (eds), AOF: realités et héritages (Dakar: Direction des Archives du Sénégal, 1997), 1151–1163 .

17 Raphaël Gallien, ‘La chair de l’asile. Le quotidien de la folie, entre violences ordinaires et ambitions discplinaires (Madagascar, 1941), *Politique Africaine*, I, 157, 71–89. Paul Marquis, *Les fous de Joinville: une histoire sociale de la psychiatrie dans l’Algérie coloniale (1933–1962)* (unpublished MA thesis: Paris Institut d’études politiques, 2021). Emmanuel Akyeampong , ‘A Historical overview of psychiatry in Africa’, in E.K. Akyeampong et al. (eds), The Culture of Mental Illness and Psychiatric Practice in Africa (Indiana University Press, 2015).

18 Some of these were opened as early as the nineteenth century. Nana Quarshie , ‘Confinement in the Lunatic Asylums of the Gold Coast from 1887 to 1906’, Psychopathologie africaine 36, 2 (2011), 191–226 .

19

Matthew M. Heaton , Black Skin, White Coats: Nigerian Psychiatrists, Decolonization, and the Globalization of Psychiatry (Ohio University Press, 2013)10.1080/13648470.2015.109660427454564, Yolana Pringle , Psychiatry and Decolonisation in Uganda (Springer Nature, 2019), Emmanuel Kwaku Akyeampong , Allan G. Hill and Arthur Kleinman , The Culture of Mental Illness and Psychiatric Practice in Africa (Indiana University Press, 2015), Katie Kilroy-Marac , An Impossible Inheritance: Postcolonial Psychiatry and the Work of Memory in a West African Clinic (University of California Press, 2019).

20

Thomas Adeyoe Lambo , ‘A Form of Social Psychiatry in Africa (with special reference to General Features of Psychotherapy with Africans’, World Mental Health 13, 4 (1961), 190–203 . Thomas Adeyoe Lambo , African Traditional Beliefs, Concepts of Health and Medical Practice. A Lecture given before the Philosophical Society. (Nigeria: University of Ibadan, 1962).

21

René Collignon , ‘Vingt ans de travaux à la clinique psychiatrique de Fann-Dakar’ dans Psychopathologie africaine 14 (1978), 133–323 .

22 Let us mention a few works which bring to light these ordinary realities starting from psychiatric institutions in post-colonial West African territories: Patrick Osouf , René Collignon & Omar Sylla , ‘Thiaroye ou les avatars d’une institution’, Psychopatrologie africaine, 13, 1 (1977), 81–111 . Danielle Stoper-Perez , La folie colonisée (Paris: Maspero, 1974). Jean-Louis, Renauld , ‘A propos de l’Assistance psychiatrique en Haute-Volta’, Psychopathologie africaine, IX, 1 (1973), 131–139 .

23

René Collignon , ‘Some Aspects of Mental Illness in French-speaking West Africa’, *in* E.K. Akyeampong et al. (eds), The Culture of Mental Illness and Psychiatric Practice in Africa (Indiana University Press, 2015), 175 .

24 We know little about the career of Charles Pidoux (1917–2004), other than that he was close to the *Évolution psychiatrique* group, general secretary of the *Société internationale d’ethnopsychologie*, and a free mason. https://www.histoiredelafolie.fr/psychiatrie-neurologie/freud-et-lethnologie-par-charles-pidoux-1955. Emmanuel Delille, ‘*De la psychiatrie exotique aux réseaux universitaires de psychiatrie*’, in H. Ellenberger (Delille ed.), *Ethno-psychiatrie* [online], Lyon: ENS Éditions, (2017), note 90. DOI: https://doi.org/10.4000/books.enseditions.7955.

25 He is clearly referred to as being the head doctor by student nurses in their dissertations throughout the 1970s. He probably, at this point, had the status of French *coopérant* doctor, as was common practice in many francophone services in the sub-region since 1960. Romain Tiquet, ‘“Tracing” Jean-Louis Renauld: Private Archives as a Way into the History of a Post-Colonial Psychiatric Unit (Bobo-Dioulasso, Upper Volta, 1972–1974)’, *Sources: Materials & Fieldwork in African Studies* [Online], 8 (2024), DOI: https://doi.org/10.4000/12n0w.

26 Pidoux Charles, ‘Etudes médico-psychologiques d’un accès dépressif à thème d’envoûtement. Contribution à la recherche ethnopsychiatrique sur les populations du Niger’, *Psyché*, 103 (1955); ‘Freud et l’ethnologie’, *Psyché*, 107–108 (1955), 477–480.

27

Jean-Pierre Olivier de Sardan , Les Sociétés Songhay-Zarma, Niger-Mali : chefs, guerriers, esclaves, paysans, (Paris, Karthala, 1984).

28 He took a particular interest in possession rituals in songhaï-zarma societies, viewing these as local institutions producing ‘a pre-therapeutic knowledge’. Charles Pidoux , ‘Les états de possession rituelle chez les Mélano-africains’, Evolution psychiatrique, 11 (1955), 271–293 13241455; ‘Les rites de possession en pays zerma (Niger)’, *Comptes-rendus des séances de l’Institut Français d’Anthropologie* (1955).

29 ‘The aim of these meetings was to produce a summary of knowledge in the field of psychology in Africa, in order to plan the (re)deployment of psychiatry and psychology for the 1960s and in light of changes connected to various countries’ Independences’, Raphaël Gallien , ‘Une psychanalyste à Madagascar. Entre histoire et anthropologie’, *in* L. Boni & S. Mendelsohn (eds), Psychanalyse du reste du monde. Géo-histoire d’une subversion (Paris, La Découverte, 2023), 179 .

30 Expert meetings of the CCTA (Commission for Technical Cooperation in Africa – reports stored in Paris at the Bibliothèque du Quai Branly for Bukavu, at the BNF for Antananarivo). Regarding these meetings and the context of the WHO’s increasing interest in ‘mental health’ in Africa, see also Yolana Pringle , ‘Negotiating South–South cooperation for mental health: The World Health Organization and the African Mental Health Action Group, 1970s–90s’, Medical History, 65, 4 (2021), 403–419 .

31 See the minutes of the conference of 5–9 March 1968 in Dakar in *Psychopathologie africaine*, IV, 1 (1968), 83–143.

32 See the minutes in *Psychopathologie africaine*, by Africano Neto for the 4th Pan-African Psychiatric Conference, XI, 2 (1975), 239–250, and by Momar Gueye for the 7th International Conference of the African Association of Psychiatrists, XIII, 2 (1977), 233–247.

33 We should note that there is no archival trace of the details of his presentations during these conferences. We can only make assumptions based on the titles of these presentations and on his published articles.

34 In his 1955 article, ‘Etudes médico-psychologiques’, Pidoux describes the trajectory of a Nigerien woman who sometimes ends up inside an institution, but the circumstances remain blurry: she is sometimes ‘brought to the infirmary’, sometimes ‘taken to a hospital room’. Ultimately, we learn that at the beginning of the 1950s she was under observation by Pidoux ‘in our service (or rather in the part of his splendid hospital which *Médecin Commandant* Mathurin [a doctor with the rank of major] had so graciously placed at our disposal)’. We can thus imagine that Pidoux was working for, or at least using the facilities of the only colonial hospital in Niger, which was managed by military staff. It did not include a psychiatric service, but French people (and their spouses, as was the case of Fatima) could probably be admitted to it for mental health issues.

35 Moustapha Louan, *Le service psychiatrique de Niamey*, final year dissertation for qualification as a registered nurse, ENSP Niamey, 1974 – 11, 19–20. The following dissertation about the service, dating back to 1976, made almost the same claim (Oumarou Elhadj Maman, *Les conditions de soins et hospitalisation des malades mentaux au pavillon E à l’hôpital de Niamey*, final year dissertation for qualification as a registered nurse, ENSP Niamey, 1976 – 2, p.12–13).

36 Pringle, *op. cit.* (note 30), 410.

37 Élodie Apard-Malah, *Queues de Pie et Grands Boubous. Une histoire franco-africaine : les relations politiques franco-nigériennes de l’après-guerre aux années 2000*, (unpublished PhD thesis: Université Panthéon-Sorbonne, Paris, 2012); Klaas Van Walraven , ‘« Opération Somme » : La French Connection et le coup d’État de Seyni Kountché au Niger en avril 1974’, Politique africaine, 134, 2 (2014), 133–154 .

38 During this period, several countries in the sub-region were corresponding with the WHO regarding the state of their hospital psychiatric services and possible new projects. See Tiquet, *op. cit.* (note 25).

39 Osouf, 1980, 258–9. This project potentially updated federal proposals and plans from the colonial period, see Aïtmedhi, *op. cit.* (note 8), 44.

40 Interview conducted by Gina Aïtmehdi with Sadio Barry, Niamey, 2011.

41 Pringle, *op. cit.* (note 30), 414. The author writes: ‘The use of Hussein Dia (…) as an AFRO consultant to advise the Niger Government on the development of a national mental health service, was an excellent example of the ‘kind of approach’ that should be ‘further followed and extended in the Region’. It was necessary not only to strengthen regional capacity, but also to confirm the value of AFRO and African expertise in international health’. The sources quoted are WHO M4/370/15BOT Jkt 2, AFRO memo from A. Franklin to N. Sartorius, 6 October 1983, 1.

42 Al Houssein Dia, *La psychiatrie au pays des marabouts* (Paris: L’Harmattan, 2008) and interview conducted by Camille Evrard with Al Houssein Dia, Nouakchott, 28 March 2022.

43 The date is confirmed by Al Houssein Dia himself, and corroborated by WHO correspondence analysed by Pringle (*op. cit.* [note 30], 414), as well as by a mention in the final year dissertation for qualification as Advanced Technician in Mental Health by Bakary Traoré in Nouakchott, *Dix ans de psychiatrie en Mauritanie* (Nouakchott: Ecole nationale supérieure des Sciences de la Santé, 1987), which specifies that, in 1983, there were less hospitalisations in the psychiatric department of the National Hospital in Nouakchott following the absence for a few months of the psychiatrist Dia, who was on assignment in Niger. Interview conducted by Camille Evrard with Al Houssein Dia, Nouakchott, 28 March 2022.

44
*Ibid.*

45
*Ibid.*

46 Dia defended his dissertation in medicine, *Une communauté thérapeutique, le Pinth de* Fann in 1972, but did not return to Mauritania until 1975, and Osouf published an article in *Psychopathologie africaine* in 1977 on the ‘forgotten’ service of Thiaroye in Senegal, based on observations made in 1975.

47 We know that he left before Barry’s arrival in September, and that Dia’s assignment took place during the rainy season. Was this Alain Bertrand, who we know supervised the following PhD thesis, which was defended in 1983 in Niamey, and who would have spent 4 years at Pavillon E? Alexis Herbert Degbey, *Contributions à l’étude des Bouffées délirantes aiguës au Niger* (unpublished PhD thesis, Niamey: Université de Niamey, 1983).

48 Oumarou Elhadj Maman, *Les conditions de soins et hospitalisation des malades mentaux au pavillon E à l’hôpital de Niamey*, final year dissertation for qualification as a registered nurse, (ENSP Niamey, 1976–2), 6–7.

49 Saint-Girons, *op. cit.* (note 8), 25.

50

Hervé Guillemain , Chronique de la psychiatrie ordinaire. Patients, soignants et institutions en Sarthe du XIXe siècle au XXIe siècle (Le Mans, Éditions de la Reinette, 2010).

51 P. Osouf states (without giving any sources for this claim) that, before the 1940s, there were secured areas or lazarets in the capital’s main healthcare centres (Osouf, *op. cit.* [note 2], 257). Saint-Girons, as well as the ENSP dissertations of Abdou Bassirou (1987–86) and Assoumana Abdou *et al.* (1989–39) refer to the years 1946–47.

52

René Collignon , ‘Pour une histoire de la psychiatrie coloniale française. À partir de l’exemple du Sénégal’, L’Autre, 3, 3(2002), 455–480 ; Romain Tiquet, ‘Gestion policière et enfermement non pénal : la mise à distance des «fous dangereux» dans le Sénégal colonial’, *Clio@Themis*, 23 (2022), *online.* DOI: https://doi.org/10.4000/cliothemis.2768

53 See Saint-Girons *op. cit.* and Aïtmehdi *op. cit.* (note 8), citing the National Archives of Niger, 1H43, ‘Internement des aliénés du Territoire du Niger’ (‘Internment of Lunatics in the Territory of Niger’).

54 Saint-Girons, *op. cit.* (note 8), 23.

55 The current Pavillon A, which explains that the office of the head doctor of the psychiatric service remained at a distance from Pavillon E for many years following its official foundation, thus reinforcing the impression of disconnection.

56 Interview conducted by Gina Aïtmehdi with Idi Kona, 2011; Saint-Girons, *op. cit.* (note 8); ENSP dissertations.

57 Interview conducted by Gina Aïtmehdi with Awa Sylla, 2011 and Sadio Barry, 2011.

58 ENSP dissertations 1987–86 and 1989–39, both mentioning interviews with Yaro Goumandeye, who at the time had just retired. Interview conducted by Gina Aïmehdi with Idi Kona, 2011.

59 Goumandeye describes frequent roundups and the presence of prisoners from the PPN-RDA. Saint-Girons, *op. cit.* (note 8), 27, 33.

60

Klaas Van Walraven , ‘Decolonization by referendum: the anomaly of Niger and the fall of Sawaba, 1958–1959’, The Journal of African History, 50, 2 (2009), 269–292 .

61 Apard, *op. cit.* (note 37).

62

Klaas Van Walraven , The yearning for relief: a history of the Sawaba movement in Niger (Leiden: Brill, 2013).

63 Our difficulties are corroborated by those of Oumy Thiongane who, while researching his PhD thesis on meningitis in Niger, came up against many grey areas in the period of the 1960s. His work informs us that in 1963, while the Health Minister of Niger was a Nigerien doctor (Harou Kouka), the Director of Public Health and the Head of the National Hospital were both French. In addition, a programme for the testing of candidate vaccines against meningitis was implemented in March 1963 using Pavillon E patients. Oumy Thiongane, *Anthropologie de la méningite au Niger. Espaces épidémiques, mobilisations scientifiques et conceptions de la maladie*, (unpublished PhD thesis: Paris, EHESS, 2013), 32–34 et 94–95.

64 Osouf, *op. cit.* (note 2), 259.

65 Saint-Girons, *op. cit.* (note 8), 26.

66 He claims to have attended a special school for nurses, midwives, and pharmacists between 1942 and 1946 under the management of Boubou Hama and a French doctor (Saint-Girons, *op. cit.* [note 8], 13–14), but this contradicts the work of Ayouba Arziza, who claims that the first training centre for nurses did not open in Niamey until 1946. It also seems difficult to reconcile with the career of Boubou Hama, who seems to have taught in Dori until 1946 before being sent off on leave by the colonial authorities for subversion (see https://bibcolaf.hypotheses.org/notices-biographiques/boubou-hama-1907-1982, viewed on 2 February 2024).

67 He tells F. Saint-Girons that his mother, Halima Chibo, was a ‘great priestess of the hawka’ and that, ‘while he did not inherit her spirits’, he did have a healer’s knowledge (p. 13 and p. 86–88).

68 We find references to Léon Tchelle in various WHO archives: he was the delegate for Niger and a doctor at Niamey hospital in 1961, and director of the National Hygiene and Mobile Medicine Service of Niger in 1968. Nothing indicates that he was a general practitioner with training in psychiatry, as is claimed in the 1976 dissertation quoted in the epigraph to the present section, written under the supervision of Pidoux, who we imagine must have known him.

69 Mahamane Barmo, *Problèmes de l’alimentation des malades mentaux internés au Pavillon E de l’hôpital de Niamey*, final year dissertation for qualification as a registered nurse, ENSP Niamey, 1976–22, 17.

70 ENSP dissertations by Moustapha Louan (1974–11) and Maman Oumarou Elhadj (1976–2).

71 ENSP dissertation by Mahamane Barmo (1976–22). In 1980, Osouf claimed that almost half the patients got food brought to them from outside. These ‘family-less’ patients were, even recently, people who had been brought in by the police and who had not been found or looked for by their parents, or those who had been left in the hands of the service.

72 Abdoulaye Bizo and Hamadou Bombou, *Les problèmes des soins des malades mentaux au Pavillon E*, final year dissertation for qualification as a registered nurse, ENSP Niamey, 1979–7, 14.

73
*Ibid*, 14–15.

74 Of this group, a vast majority was classified in the category of ‘indigents’. We can follow the evolution of prisoners hospitalised on the Pavillon E level: there were around a hundred of them per year during the second half of the 1960s, then they almost disappeared towards 1978, but two students noted in 1980 and 1981 that the ‘security/prison’ service did indeed exist, catering to 16 prisoners in February 1980.

75 For the purposes of comparison, in the last dissertation we found, dating from 1992, the student mentions 60 patients when they arrived/upon their arrival in/at the service.

76 Osouf, *op. cit.* (note 2), 261.

77

Patricia Rossat , ‘La psychiatrie au Niger’, Psychopathologie africaine, XVI, 3, (1980), 281–284 .

78 Saint-Girons, *op. cit.* (note 8), 27–30.

79 This is Osouf’s expression – he mentions research in the field of anthropology, and calls for a public debate on the topic in order to advance mental health care in Niger. If he ever did get the opportunity to discuss this with Major Goumandeye, he does not mention it. Osouf, *op. cit.* (note 2), 263–4.

80 The use of this term is long-established, which shows the importance given to putting mentally ill patients to work, as early as in the thinking of Reboul and Régis, ‘L’assistance des aliénés aux colonies’, *Congrès des médecins aliénistes de France et des pays de langue française* (Paris: Masson ed.,1912).

81 Saint Girons, *op. cit.* (note 8), 30: the nurse seems to have also organised the ‘mad people’s market’, small stalls offering everything and nothing, first within the hospital and then outside of it; he also used some patients to work in his own fields, and others were almost integrated into his family. The ENSP dissertations also note the participation of charity organisations in ergotherapy, cooking, and sewing workshops.

82 At the initiative of a female nurse, explains ENSP dissertation 1976–2. See also Brossat, *op. cit.* (note 77), 283.

83 Brossat, *op. cit.* (note 77), 282–284.

84 See ENSP dissertation 1981–13 and the interview conducted by Gina Aïtmehdi with Awa Sylla, 2011.

85

Gina Aïtmehdi , ‘Psychiatrie et chronicités au Niger. Négocier les responsabilités du soin dans la maldie au long cours’, Emulations, 27 (2108), 47–59 .

86 Reforming figures, or figures of precursors, which are the product of their time, are often called upon in contexts close to the local oral history of psychiatry, with varying degrees of unanimity in terms of the discourse related to them. We are thinking here in particular of Henri Collomb in Senegal, Al Hussein Dia in Mauritania, Jean-Luc Coudray and Baba Koumaré in Mali, or Jean-Louis Renaud in Bobo-Dioulasso.

87 Term taken from an interview with Doctor Barry on 22 October 2011, and also used in an interview with Awa Sylla, a nurse who worked at Pavillon E between 1982 and 1988, on 22 November 2011. These interviews were conducted by Gina Aïtmehdi.

88 Interview conducted by Gina Aïtmehdi with Awa Sylla, 2011.

89
*Ibid.*

90

Anne M. Lovell , Ursula M. Lire & Claudia Lang , ‘Genealogies and Anthropologies of Global Mental Health’, Culture, Medicine, and Psychiatry, 43 (2019), 519–547 31729686 10.1007/s11013-019-09660-7.

91 Interview conducted by Gina Aïtmehdi with Doctor Barry, 2011.

92

Nicolas Henckes , ‘Reshaping chronicity : neuroleptics and changing meanings of therapy in French psychiatry, 1950–1975’, Studies in History and Philosophy of Biological and Biomedical Sciences, 42 (2011), 434–442 22035717 10.1016/j.shpsc.2011.05.004. Hervé Guillemain , ‘Les effets secondaires de la technique. Patients et institutions psychiatriques au temps de l’électrochoc, de la psychochirurgie et des neuroleptiques retard (années 1940–1970)’, Revue d’Histoire Moderne & Comptemporaine, 67, I (2020), 72–98 .

93 Extract from an interview conducted by Gina Aïtmehdi with a nurse, Idi Kona, who arrived at the service in 1986.

94 Interview conducted by Gina Aïtmehdi with Awa Sylla, 2011.

95 Gina Aïtmehdi, *op. cit.* (note 85).

96

Babakar Diop and Maurice Dorès , ‘L’admission de l’accompagnement du malade à l’hôpital psychiatrique’, Perspectives Psychiatriques, 59, 5 (1976), 359–368 .

97 This secondary benefit of a standard which was initially established for clinical reasons is reflected in other contemporary contexts. On this subject, see Katie Kilroy-Marac , ‘Of shifting economies and making ends meet: The changing role of the *accompagnant* at the Fann psychiatric clinic in Dakar, Senegal’, Culture, Medicine, and Psychiatry 38, 3 (2014), 427–447 25034610 10.1007/s11013-014-9386-7.

98 Interview conducted by Gina Aïtmehdi with Awa Sylla, 2011.

99 Gina Aïtmehdi, *op. cit.* (note 85).

100 Interview conducted by Gina Aïtmehdi with Awa Sylla, 2011.

101
*Ibid.*

102 Alongside this work, we launched a project to collect, sort, classify, and preserve all the archives in Pavillon E. Supported by department head Dr Oumou Ousseyni Zika and financed by the ERC MadAf project, it was carried out in July–August 2024 under the direction of archivists Harouna Maurice Diallo and Adamou Amadou, with logistical coordination by Ousmane Kansaye. This completed project is sure to give rise to further work. See https://madaf.hypotheses.org/programme-de-conservation-des-archives-psychiatriques-du-pavillon-e-de-lhopital-national-de-niamey-niger

